# Mummy of a juvenile sabre-toothed cat *Homotherium latidens* from the Upper Pleistocene of Siberia

**DOI:** 10.1038/s41598-024-79546-1

**Published:** 2024-11-14

**Authors:** A. V. Lopatin, M. V. Sotnikova, A. I. Klimovsky, A. V. Lavrov, A. V. Protopopov, D. O. Gimranov, E. V. Parkhomchuk

**Affiliations:** 1 Borissiak Paleontological Institute, Russian Academy of Sciences, Moscow, Russia; 2 Geological Institute, Russian Academy of Sciences, Moscow, Russia; 3 Academy of Sciences of the Republic of Sakha (Yakutia), Yakutsk, Russia; 4 Institute of Plant and Animal Ecology, Ural Branch of the Russian Academy of Sciences, Ekaterinburg, Russia; 5 Novosibirsk State University, Novosibirsk, Russia

**Keywords:** Palaeontology, Ecology, Evolution, Zoology

## Abstract

The frozen mummy of the large felid cub was found in the Upper Pleistocene permafrost on the Badyarikha River (Indigirka River basin) in the northeast of Yakutia, Russia. The study of the specimen appearance showed its significant differences from a modern lion cub of similar age (three weeks) in the unusual shape of the muzzle with a large mouth opening and small ears, the very massive neck region, the elongated forelimbs, and the dark coat color. Tomographic analysis of the mummy skull revealed the features characteristic of Machairodontinae and of the genus *Homotherium*. For the first time in the history of paleontology, the appearance of an extinct mammal that has no analogues in the modern fauna has been studied.

## Introduction

In 2020, the frozen mummified carcass of a large carnivore cub was found in the Abyisky ulus of the Republic of Sakha (Yakutia). The locality, called Badyarikhskoe, is located on the Badyarikha River (right tributary of the Indigirka River, Yana-Indigirka Lowland; 67°41ʹ14ʹʹN, 146°46ʹ13ʹʹE). The numerous bones of mammoth fauna representatives are collected from the loess-like loams of the Yedoma horizon in this locality.

Radiocarbon dating of the find (based on wool) is 31,808 ± 367 years BP, calibrated (based on the Intcal20 calibration curve in the OxCal 4.4 program) as 35,471–37,019 years cal BP (probability 95.4%; CCU AMS NSU-NSC, no. GV-4961, accelerator mass spectrometer MICADAS-28).

Findings of frozen mummified remains of the Late Pleistocene mammals are very rare. In Russia, the most of these finds are concentrated in the Indigirka River basin. Over the past 10 years, mummies of various animals were discovered there^1,2^.

**Fig. 1:**
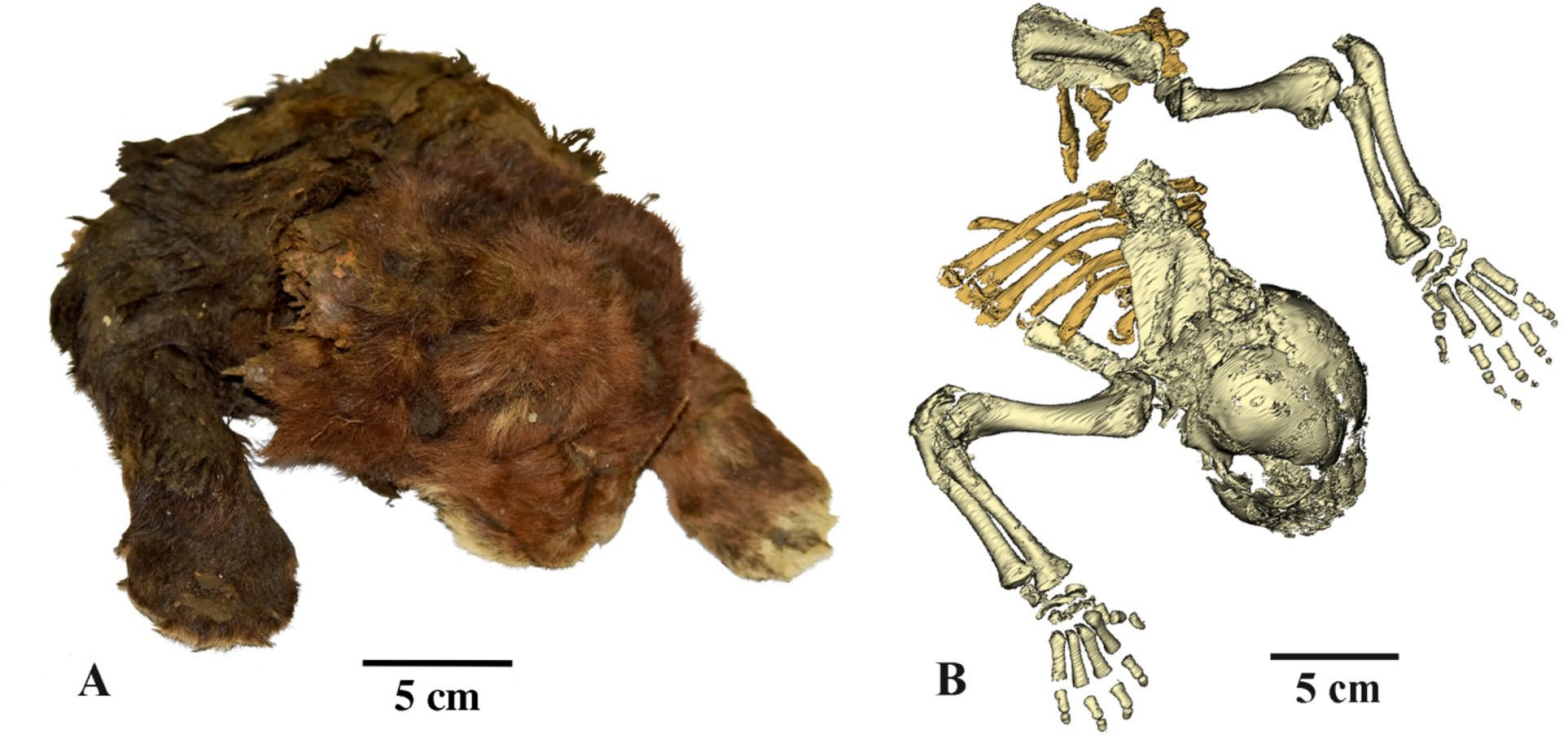
The frozen mummy of *Homotherium latidens* (Owen, 1846), specimen DMF AS RS, no. Met-20-1, Russia, Republic of Sakha (Yakutia), Indigirka River basin, Badyarikha River; Upper Pleistocene: (**A**) external appearance; (**B**) skeleton, CT-scan, dorsal view.

The Badyarikha mummy (specimen DMF AS RS, no. Met-20-1) contains the head and the anterior part of the body preserved approximately to the caudal edge of the chest (Fig. 1). There are also incomplete pelvic bones articulated with the femur and shin bones. They were found encased in a piece of ice along with the front part of the cub corpse. The specimen is stored at DMF AS RS in Yakutsk.

The study of the mummy was carried out at the PIN. The carcass of a three-week-old lion cub, *Panthera leo* (Linnaeus, 1758), from the collection of the ZMMU (no. S-210286) was used for comparative analysis. In addition, skulls of juvenile *P. leo* from the ZMMU collection (nos. S-3000, S-3034, S-3065, S-3066–3068) were compared. The skeleton structure of the Badyarikha mummy was examined using the Siemens Somatom go.Up CT scan (Skolkovo Vet Veterinary Hospital, Moscow). To work with the resulting slices, the programs RadiAnt DICOM Viewer 2022.1.1 and Blender 3.6.4, 3D Slicer 5.6.0 were used.

We focused on the analysis of the cranial and dental features of the mummy to determine its taxonomic position. Among the postcranial elements, we analyzed here only the most striking morphological peculiarities determined visually. The anatomical features of the find will be discussed in more detail in a subsequent publication.

Measurements were made according to standard techniques. All measurements are given in mm. External measurements of *P. leo* are given according to specimen ZMMU, no. S-210286. Skull measurements of juvenile *P. leo* are given based on specimens ZMMU, nos. S-3066 (two weeks) and S-3034 (three weeks). Measurements of the mummy skull and teeth were made using the 3D computer model. For lateromedial skull sizes, measurements of the undeformed right part of the skull were multiplied by 2. For length measurements of cranial elements, readings were taken from the undeformed right side of the skull.

Abbreviations: H, height; L, length; L1, condylobasal length; W, width. Institutions: CCU AMS NSU-NSC, Center for Collective Use of the Accelerator Mass Spectrometry Complex at the Novosibirsk State University and Novosibirsk Scientific Center, Novosibirsk, Russia; DMF AS RS, Department for the Study of Mammoth Fauna, Academy of Sciences of the Republic of Sakha (Yakutia), Yakutsk, Russia; PIN, Borissiak Paleontological Institute, Russian Academy of Sciences, Moscow, Russia; UrFU, Ural Federal University named after the First President of Russia B.N. Yeltsin, Ekaterinburg, Russia; ZMMU, Zoological Museum of Moscow State University, Moscow, Russia.

## Description and comparison

Judging by the eruption stage of the deciduous incisors^3,4^, the Badyarikha mummy belongs to the same age group as a three-week-old lion cub, ZMMU S-210286.

The number of ribs that have been established for the mummy approximately corresponds to the full length of the thoracic spine. Six pairs of ribs in anatomical order are clearly visible (Fig. 1, B). Several fragments of ribs are also visible here. The remaining fragments of ribs and vertebrae have not yet been separated from the total mass of soft tissues and bones. According to our estimate, their number corresponds to 12 pairs of ribs. This is almost equal to the length of the thoracic region of Felidae (13 vertebrae and pairs of ribs). On this basis, an estimated comparison of the body sizes of the mummy and juvenile *P. leo* was carry out.

The length of the preserved part of the mummy body from the nose tip to the gap in the chest region (at the level of the 12th vertebra) is 248.0. A similar length in *P. leo*, specimen ZMMU S-210286 is 273.0 (total body length, 355.0). The length of the mummy head to the visual border of the occipital region is 108.0 (92.0 in ZMMU S-210286). Thus, the length of this body part of specimen DMF AS RS, no. Met-20-1 is only 10% less than that of *P. leo*, specimen ZMMU S-210286.

The facial region length from the DI1 alveolus to the line of the dorsomedial edge of the auricle of the ear in the mummy is 85.0 (83.0 in ZMMU S-210286). The facial region length from the nose tip to the anterior edge of the eye slit is 32.0 and 40.0, respectively.

**Fig. 2:**
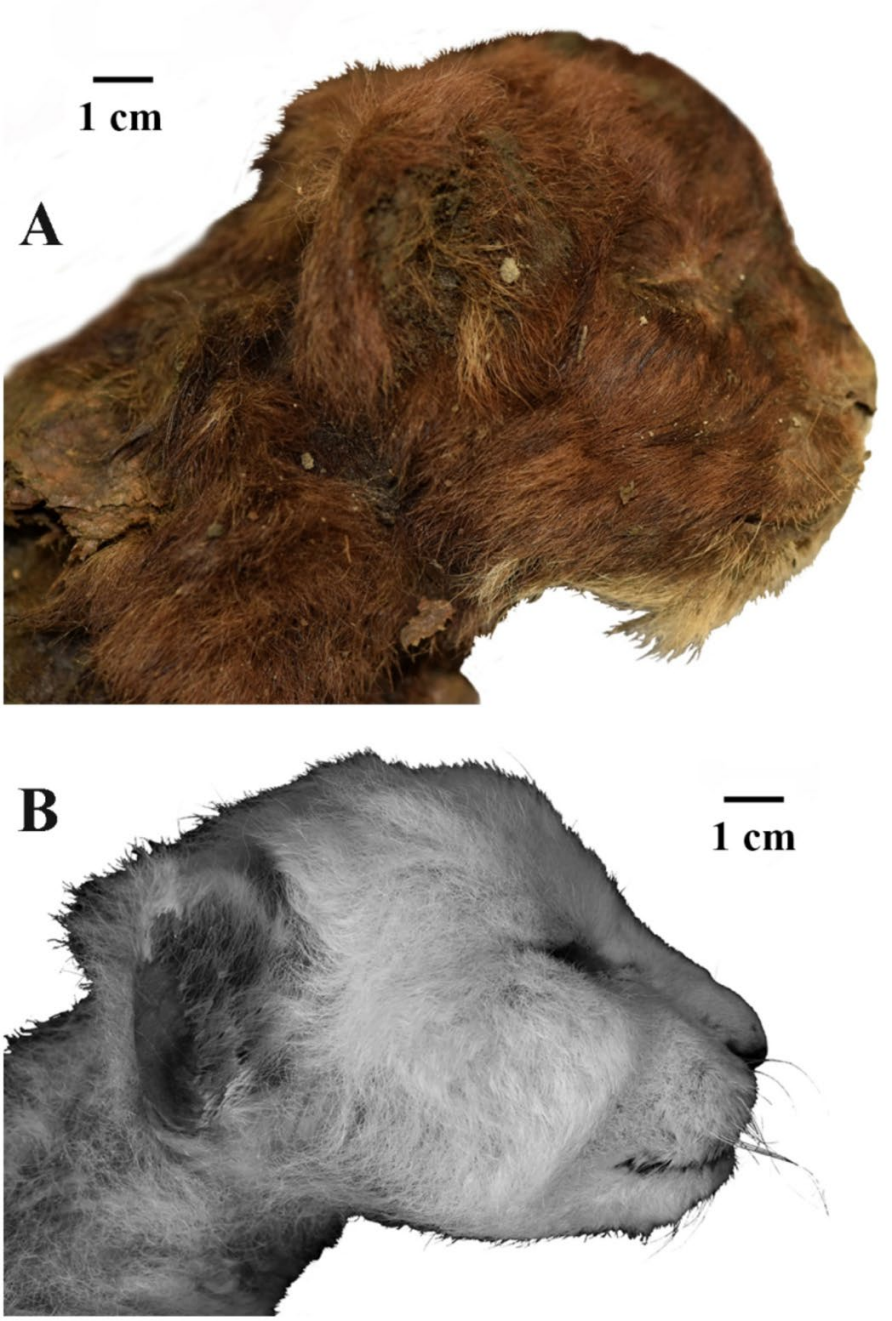
External appearance of three-week-old heads of large felid cubs, right lateral view: (**A**) *Homotherium latidens* (Owen, 1846), specimen DMF AS RS, no. Met-20-1, frozen mummy, Russia, Republic of Sakha (Yakutia), Indigirka River basin, Badyarikha River; Upper Pleistocene; (**B**) *Panthera leo* (Linnaeus, 1758), specimen ZMMU, no. S-210286; Recent.

The mummy body is covered with short, thick, soft, dark brown fur with hair about 20–30 mm long. The fur on the back and neck is longer than on the legs. On the upper lip two rows of vibrissae are clearly visible, mostly broken off at a height of 3–5 mm from the roots. In the region of the mouth corner, the hair is significantly elongated (Figs. 1 and 2).

The mummy head is well preserved. In the dorsal projection, the eye slits of the mummy are oriented at the angle of approximately 45º to the sagittal axis of the skull. In *P. leo* (ZMMU S-210286), the eye slits are located at the angle of about 90º to the sagittal axis; their position almost coincides with the frontal plane. We assume that the difference in the position of the eyes on the skull is due to postmortem deformation of the soft tissues. The eye slit in the mummy is slightly longer than in ZMMU S-210286. The mummy eyelashes were not preserved.

The mummy auricle has the shape of a low semicircular arch. In contrast to that of *P. leo* (ZMMU S-210286), it slightly protrudes beyond the contour of the head (it strongly protrudes in ZMMU S-210286). In the mummy, the height of the auricle is 3.6 times less than the width (15.0 vs. 54.0; in ZMMU S-210286, height is 28.0, width is 29.0). The ear is located quite high on the skull; its ventrolateral edge is at the level of the upper dentition (in ZMMU S-210286, it is much lower, slightly above the ventral edge of the mandible).

The shapes of the nasal planum and nostrils of the mummy are typical to Felidae. The height of the nasal planum is 15.0; its width (between the lateral edges of the nostrils) is 26.0. The nostril openings are located within the nasal planum. In ZMMU S-210286, the external edges of the nostril openings extend beyond the nasal planum by 3 mm, the width of the nasal planum is 20.1. The upper lip height in the mummy (7 mm) exceeds that of the lion cub more than twice (in ZMMU S-210286, this height is 3.1) The upper lip height of the mummy was estimated by the distance from the junction of the soft tissues with the maxilla to the ventral lip edge. Obviously, this difference is due to the further ontogenetic development of the long upper canine and the need to cover it with an upper lip. The width of the right part of the oral fissure from the sagittal axis is 37.0, the width of the left part is 34.5 (difference due to the deformation of the skull). The measurement points of the oral opening: a point at the notch of the upper lip on the sagittal axis and a point at the mouth corner. In the juvenile *P. leo*, the length of the oral fissure (measured in a similar way) is 31.0 mm. Therefore, in the *Homotherium* mummy, this measurement is larger by 11.3–19.3%. On average, the oral fissure size of the mummy is larger by 15.3%.

The mummy neck is longer and more than twice as thick as that of *P. leo*, ZMMU S-210286 (80.0 vs. 74.0, 52.0 vs. 32.0, respectively). The difference in thickness is explained by the large volume of muscles, which is visually observed at the site of separation of the skin from the mummified flesh.

**Fig. 3:**
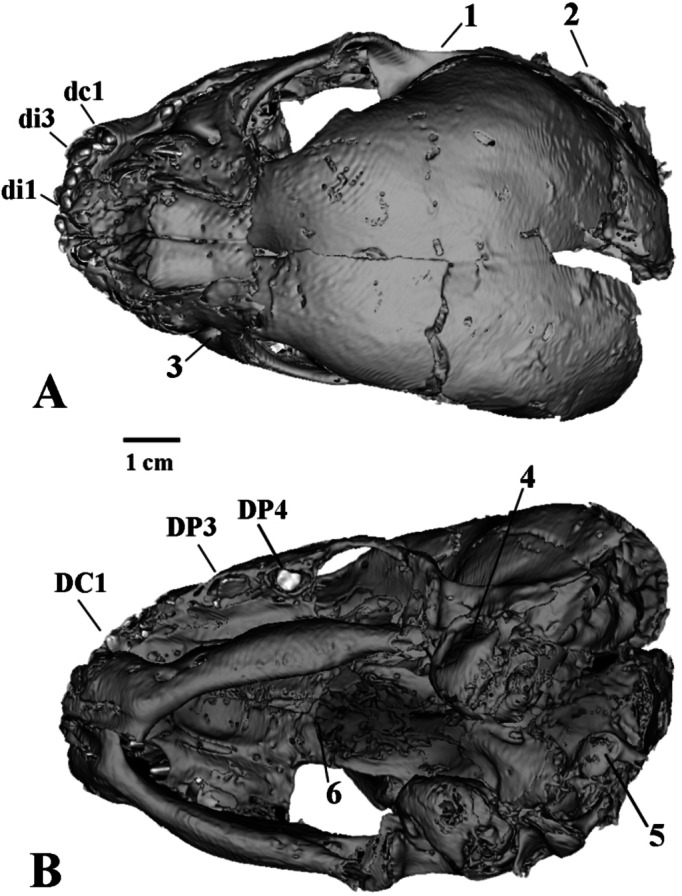
The deformed mummy skull with the lower jaw of *Homotherium latidens* (Owen, 1846), specimen DMF AS RS, no. Met-20-1, Russia, Republic of Sakha (Yakutia), Indigirka River basin, Badyarikha River; Upper Pleistocene; CT-scan: (**A**) dorsal view; (**B**) ventral view. Designations: 1, suprameatal crest; 2, mastoid process; 3, infraorbital foramen; 4, external auditory meatus; 5, occipital condyle; 6, internal nares; unerupted lower deciduous teeth: di2–di3, incisors; dc1, canine; unerupted upper deciduous teeth: DC1, canine; DP3–DP4, premolars.

**Fig. 4:**
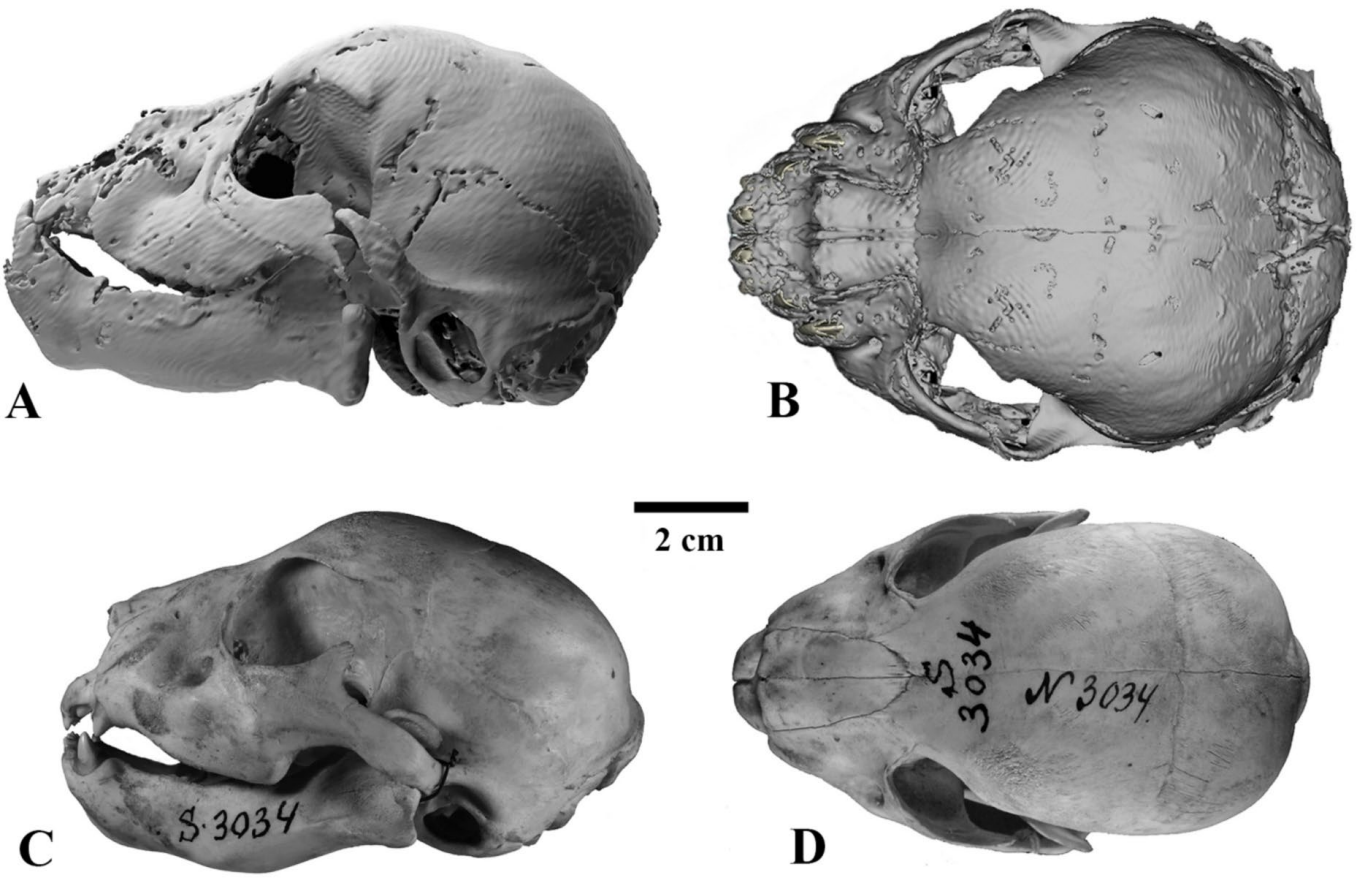
Skulls of three-week-old large felid cubs, left lateral view (**A**, **C**) and dorsal view (**B**, **D**): A, B, *Homotherium latidens* (Owen, 1846), specimen DMF AS RS, no. Met-20-1, frozen mummy, 3D computer models (image is reconstructed based on the undeformed right half of the skull, mirrored); Russia, Republic of Sakha (Yakutia), Indigirka River basin, Badyarikha River; Upper Pleistocene; C, D, *Panthera leo* (Linnaeus, 1758), specimen ZMMU, no. S-3034, photographs; Recent.

**Fig. 5:**
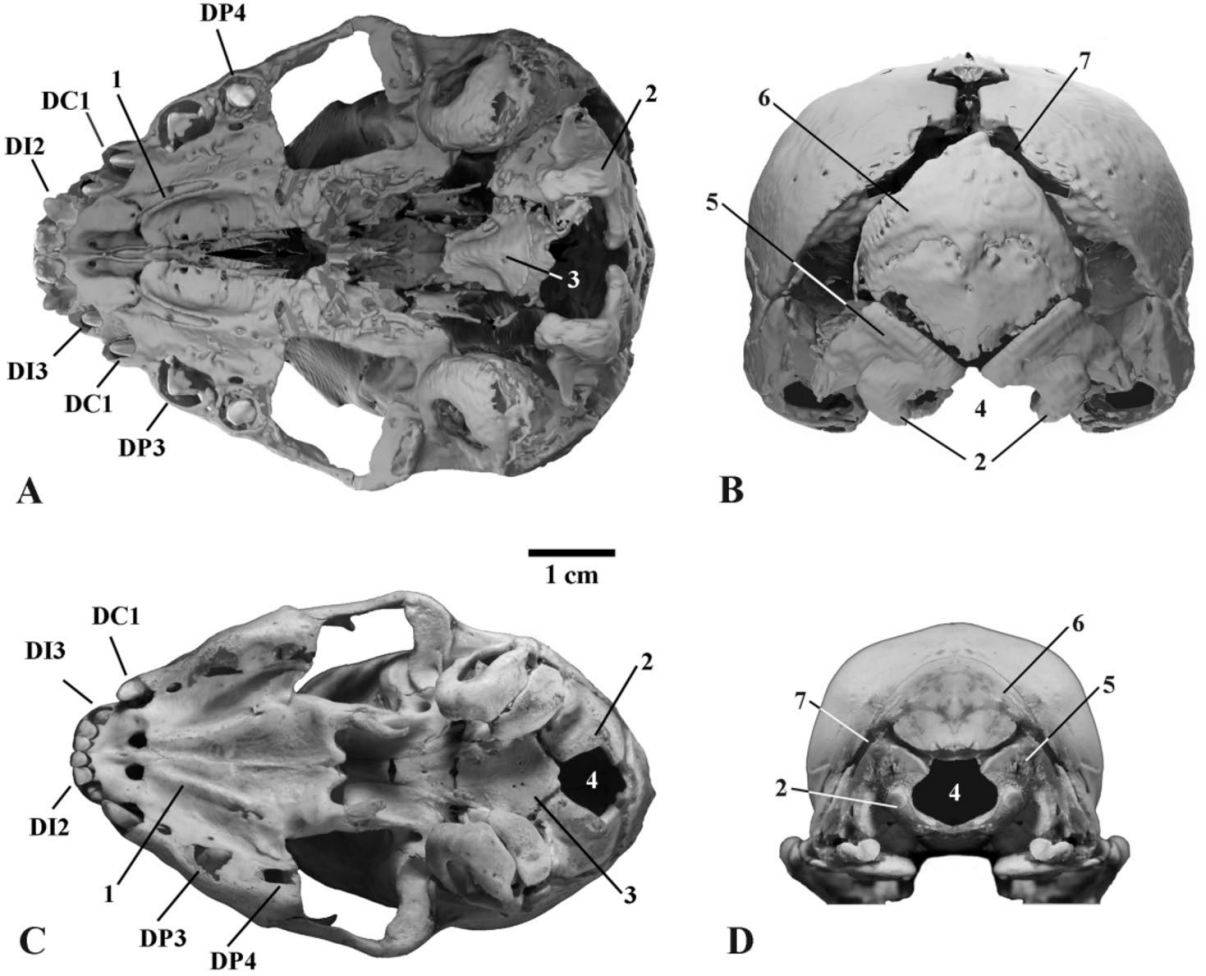
Skulls of three-week-old large felid cubs, ventral view (**A**, **C**) and occipital view (**B**, **D**): A, B, *Homotherium latidens* (Owen, 1846), specimen DMF AS RS, no. Met-20-1, frozen mummy, 3D computer models (image is reconstructed based on the undeformed right half of the skull, mirrored); Russia, Republic of Sakha (Yakutia), Indigirka River basin, Badyarikha River; Upper Pleistocene; C, D, *Panthera leo* (Linnaeus, 1758), specimen ZMMU, no. S-3034, photographs; Recent. Designations: 1, palatine ridges; 2, occipital condyle; 3, basioccipital; 4, foramen magnum; 5, exoccipital; 6, occipital; 7, occipital-parietal suture; unerupted upper deciduous teeth: DI2–DI3, incisors; DC1, canine; DP3–DP4, premolars.

**Fig. 6:**
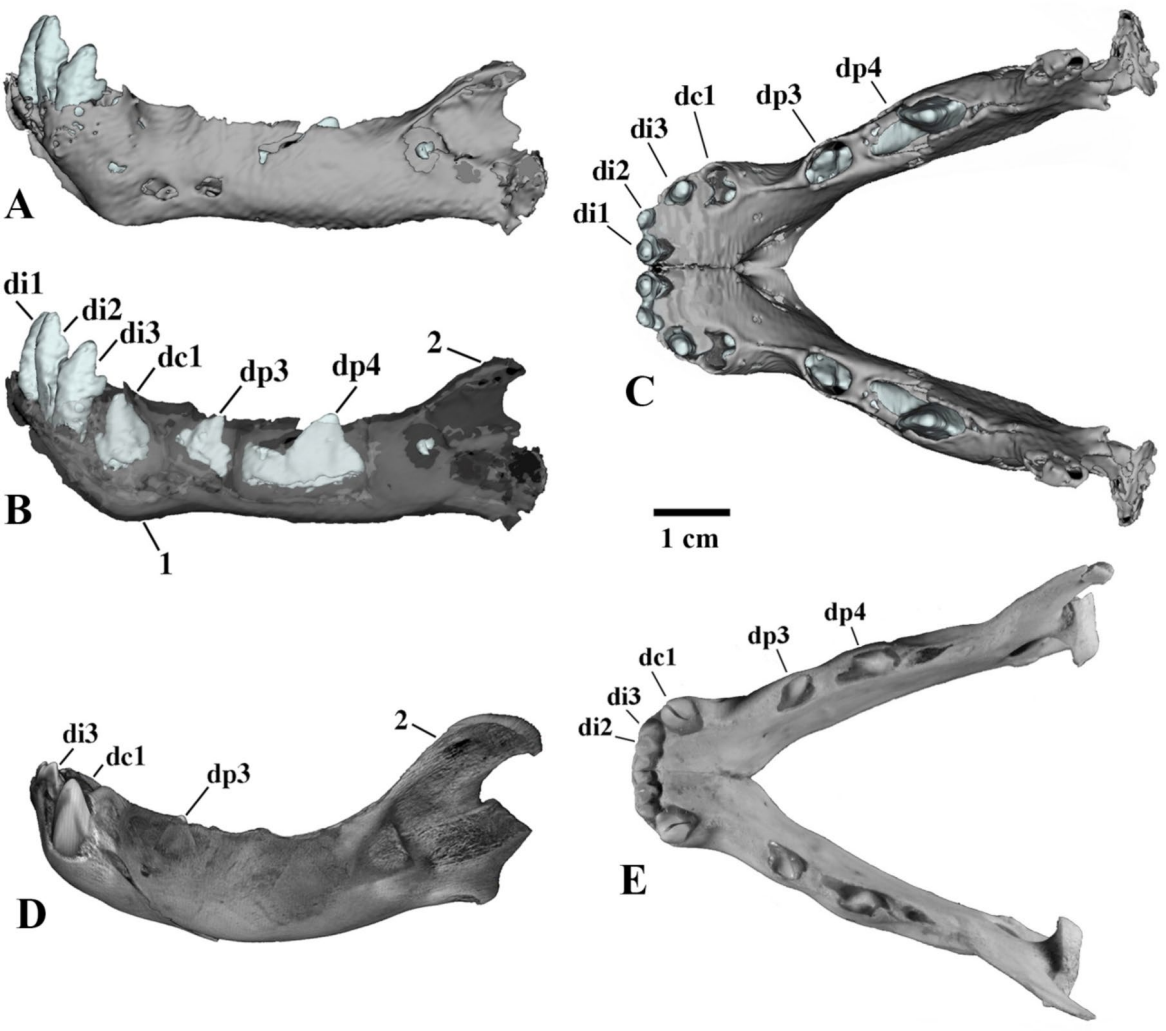
Mandibles of three-week-old large felid cubs, left lateral view (**A**, **B**, **D**) and occlusal view (**C**, **E**): A–C, *Homotherium latidens* (Owen, 1846), specimen DMF AS RS, no. Met-20-1, frozen mummy: A, C, 3D computer model (C, image is reconstructed based on the undeformed right ramus of the mandible, mirrored); B, parasagittal section; Russia, Republic of Sakha (Yakutia), Indigirka River basin, Badyarikha River; Upper Pleistocene; D, E, *Panthera leo* (Linnaeus, 1758), specimen ZMMU, no. S-3034, photographs; Recent. Designations: 1, mandibular flange; 2, coronoid process; unerupted lower deciduous teeth: di1–di3, incisors; dc1, canine; dp3, and dp4, premolars.

The general morphology of the skull is typical of juvenile Felidae (Figs. 3, 4, 5 and 6). We establish that the mummy belongs to the genus *Homotherium* Fabrini, 1890 on the base of a series of morphological features of the lower jaw: a pronounced mandibular flange, a short and low coronoid process, a deep symphysis region and an elevated position of the incisors relative to the cheek teeth row (Fig. 6). Together with the canine, the deciduous lower incisors form a single arched row, in contrast to *P. leo* (ZMMU S-210286). The mummy skull is also distinguished by a relatively longer facial region, a rounded braincase, expanded zygomatic arches, a wide area of premaxillaries, and large upper deciduous incisors (Figs. 4 and 5).

The mummy skull length (L1, 102.2) is 10% greater than that of the ZMMU S-3034 (L1, 92.3), and 10–18% greater than those of other compared juvenile *P. leo* from the ZMMU collection (L1, 86.5–92.3; *n* = 3). The skull is moderately deformed (Fig. 3). Its shape was reconstructed on the basis of the well-preserved right half (Figs. 4, 5 and 6).

The nasal bones of the *Homotherium* cub compared to *P. leo* (ZMMU S-3034) are greatly shortened and widened (Table 1). The suture between the nasal and frontal bones looks like a straight line, in contrast to the U-shaped suture of ZMMU S-3034.

**Table 1:** Skull measurements of juvenile *Homotherium latidens* (Owen, 1846) and *Panthera leo* (Linnaeus, 1758).

Parameter	*H. latidens*, specimen DMF AS RS, no. Met-20-1	*P*. *leo*, specimen ZMMU, no. S-3066	*P*. *leo*, specimen ZMMU, no. S-3034	Ratio of the skull size of *H. latidens* to the skull size of *P*. *leo*, specimen ZMMU, no. S-3034, %
Individual age	3 weeks	2 weeks	3 weeks	–
Condylobasal length of the skull (mm)	102.2	74.1	92.3	110.7
Maximum width of the braincase (mm)	67.9	46.6	54.2	125.3
Mastoid width (mm)	70.5	41.6	46.5	151.6
Maximum maxillary width (at alveolar level of DP4) (mm)	63.4	52.3	50.7	125.0
Width of the upper row of incisors (mm)	28.3	13.5	16.1	175.8
Length of the facial region (mm)	33.3	20.8	28.1	118.5
Length of the nasal bones (mm)	18.5	19.6	28.0	66.1
Length of the mandible (mm)	72.7	54.6	66.7	109.0
Depth of the mandible at the base of the coronoid process (mm)	13.1	10.6	14.8	88.5
Depth of the mandible including the coronoid process (mm)	20.9	20.3	23.2	90.1

In the mummy, the infraorbital foramen is located mesial to the anterior margin of the orbit, it is slit-like, oriented dorsoventrally, and its long axis is inclined at approximately 30º to the horizontal plane. In *P. leo* (ZMMU S-3034), the infraorbital foramen is located directly under the anterior edge of the orbit; its shape is almost round.

The zygomatic arches of juvenile *Homotherium*, in contrast to *P. leo*, are spaced more widely (although in adult *Homotherium*^5^ the width of the skull in the zygomatic arches is much smaller than in Pantherinae).

The braincase of the *Homotherium* cub mummy is swollen, greatly expands caudally, its volume is larger than that of *P. leo* (ZMMU S-3034). The mastoid width of the braincase of the mummy is 70.5; in the compared specimen of *P. leo*, mastoid width is 41.6. The occipital bones of juvenile *Homotherium* are separated by wide fontanelles. These include the unfused left and right occipitals and interparietal bone, which is typical of two-week-old *P. leo* cubs (these bones are fused at the age of three weeks in lions). The axis of the foramen magnum in the described cub of *Homotherium* is oriented dorsoventrally, in contrast to juvenile Pantherinae (in *P. leo*, it is oriented mesiodistally even at the age of two weeks). The maximum width of the braincase, mastoid width, upper incisor row width, and facial region length in juvenile *Homotherium* are disproportionately greater than in same-aged (three-week-old) *P. leo*. At the same time, length of the nasal bones, depth of the horizontal ramus of the mandible and height of the coronoid process are disproportionately smaller in *Homotherium*.

The hard palates of the skulls of the juvenile *Homotherium* and *Panthera leo* (Fig. 5) exhibit two parasagittally located bony “palatine ridges” (sensu^6^: p. 6, Fig. 5, D, E). These palatine ridges are preserved in adult *Homotherium*,* Smilodon* and other Machairodontinae in contrast to Pantherinae members^6,7^. This fact indicates that the presence of the palatine ridges in adult Machairodontinae is retention of the pedomorphic character.

One of the striking features of the morphology of *Homotherium*, both in adults and in the studied cub, is the presence of an enlarged premaxillary bone, containing a lateromedially expanded row of large cone-shaped incisors that form a convex arch. Among all the unerupted teeth of the *Homotherium* cub mummy, only the upper and lower deciduous incisors protrude with their tops from the alveoli. The sizes of the incisors increase from DI1/di1 to DI3/di3; all incisors have two accessory cusps, a large distal and a smaller mesial one. Serration on the side ridges of the incisors is not visible and cannot be felt during palpation. The 3D computer model shows the pointed tips of the highly flattened upper deciduous canines, also without serrations. This is quite unusual. Probably, the CT-scan resolution power is too low to register such small structures. The tips of the lower canines dc1 are visible in the open alveoli; dc1 has a large mesial cusp. In the occlusal view, it is clearly visible that the lower deciduous canine is included in the incisor row.

The deciduous cheek teeth and canines of the *Homotherium* cub mummy are formed to the hollow cap stage. Unlike DP3, the crown of DP4 is fully formed. DP4 is small, round in occlusal view, with an almost flat occlusal surface. In *Homotherium crenatidens* fabrini, 1890, DP4 is tribosphenic and similar to DP4 of Pantherinae^8^. The dp3 shows a four-cusped structure typical of *Homotherium* deciduous teeth. The main cusp is high, the anterior and first posterior cusps are equally developed. The second posterior cusp is present, but not yet fully formed. There are dp3 and dp4 of *Homotherium* from the Early Pleistocene localities of Europe, Fonelas P-1 in Spain, Pirro Nord (Pirro 10) in Italy, and Untermassfeld in Germany^9–11^ and from the Late Pleistocene of North America^4^. The morphology and dimensions of the Badyarikha mummy dp3–dp4 are close to those of *Homotherium* from the mentioned localities. Minor differences are observed in the structure of dp4. In European forms, the dp4 talonid is better individualized and compact, in contrast to the more elongated metaconid–talonid region of dp4 in the mummy.

Measurements of deciduous teeth of the mummy: DI1: L, 3.7; W, 3.5; DI2: L, 5.6; W, 4.2; DI3: L, 5.8; W, 7.5; DC1: L, 6.5; W, 3.0; DP3: L, 13.2; W, 2.7; DP4: L, 4.6; W, 4.5; di1: L, 3.0; W, 3.2; di2: L, 4.4; W, 4.5; di3: L, 4.5; W, 5.8; dc1: L, 7.3; W, 4.1; dp3: L, 7.5; W, 3.7; dp4: L, 15.8; W, 5.0.

The forelimbs of the mummy were preserved almost completely (Fig. 1). The left limb is skeletonized above the elbow joint. Partial soft tissue rupture in the area of the wrist joint makes it possible to determine the length of the forearm based on the ulna; it is 94.4. The length of the right forelimb measured from the posterior edge of the olecranon to the base of the claw of the third finger is 154.8. The humerus length (measured directly) is 75.6, the width of the distal epiphysis is 22.5, the width of the proximal epiphysis is 26.9 (in ZMMU S-210286, forelimb length is 125.4 and humerus length is 64.3).

**Fig. 7:**
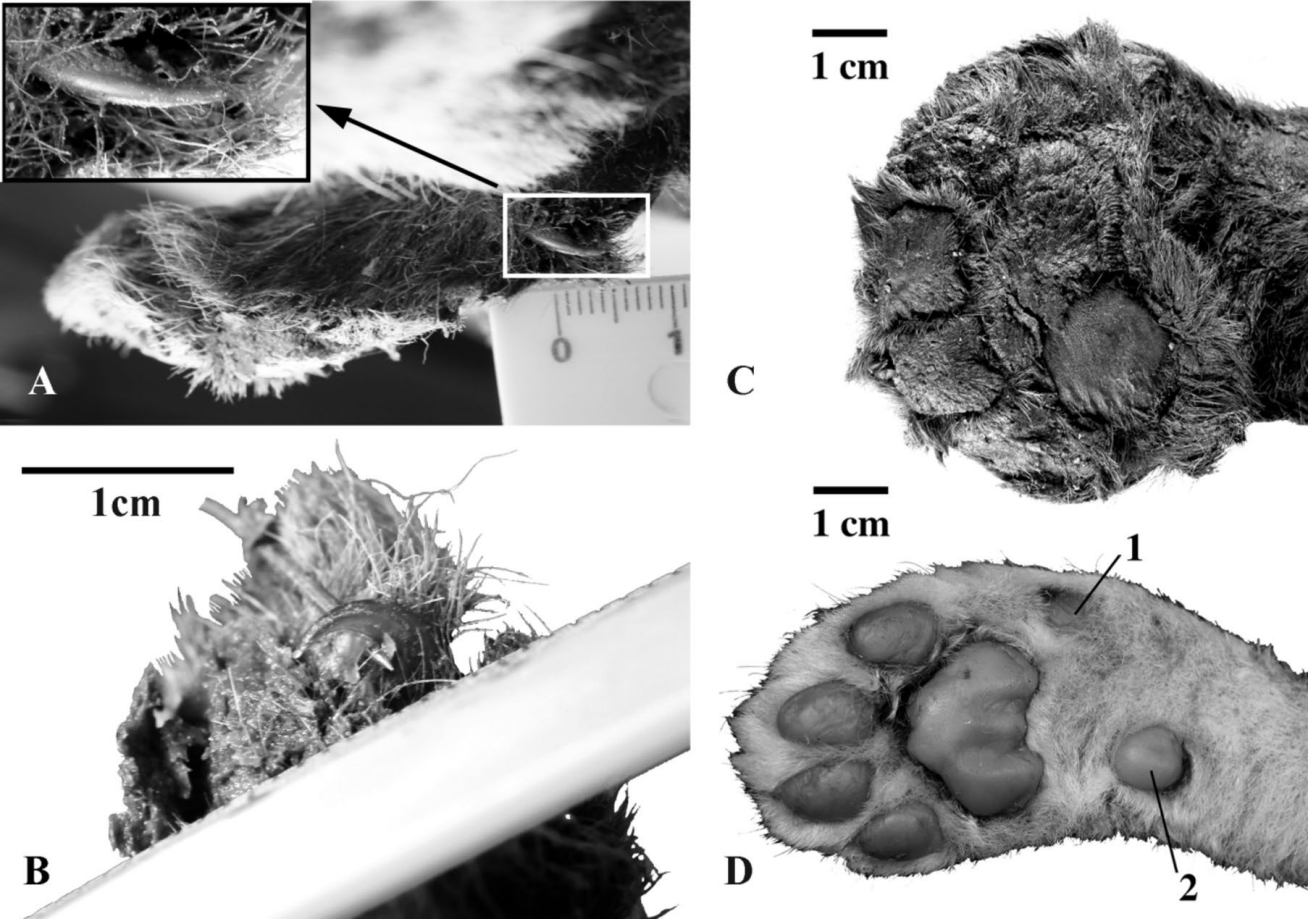
Forepaws of three-week-old large felid cubs: **A**, **B**, **С**, *Homotherium latidens* (Owen, 1846), specimen DMF AS RS, no. Met-20-1, frozen mummy, right forepaw; Russia, Republic of Sakha (Yakutia), Indigirka River basin, Badyarikha River; Upper Pleistocene: A thumb claw; B second digit claw; С plantar view; (**D**) *Panthera leo* (Linnaeus, 1758), specimen ZMMU, no. S-210286, right forepaw, plantar view; Recent. Designations: 1, first digital pad; 2, carpal pad (absent in *H. latidens*).

The foot pads and claws are preserved on the plantar surface of the front paw of the *Homotherium* cub mummy (Fig. 7). The length of the left front paw (to the scapholunatum – radius line) is 62.5; the width excluding the thumb, 51.1; the width including the thumb, 57.3. The length of the autopodium along the plantar side to the visually traced line of the wrist joint is 55.0 (59.4 in ZMMU S-210286). The mesiodistal length of the wrist joint is 29.4, its lateromedial width is 40.4. The absence of a carpal pad is established for the mummy front paw. The digital pads of the first and fifth digits of the mummy have the shape of a right triangle. The long side of the fifth digital pad is adjacent to the fourth digital pad. The shape of the second–fourth digital pads is subsquare, in contrast to the oval digital pads of the lion and other Felidae. The metacarpal pad is bilobed and bean-shaped.

Dimensions (L/W) of the metacarpal pad of the right front paw are 29.4/40.4 (in ZMMU S-210286, 15.0/20.3). Dimensions (L/W) of the digital pads: I, 9.1/6.5; II, 14.7/13.7; III, 13.4/14.3; IV, 14.1/14.0; V, 13.9/10.1 (in ZMMU S-210286, 5.0/4.8, 9.6/6.9, 10.7/6.9, 10.1/6.3, 9.4/6.2, respectively).

All claws are preserved on the fingers. They are sharp and strongly curved (Fig. 7). In cross section, the claws are laterally compressed and have the same shape as in *P. leo* (ZMMU S-210286). The length of the thumb claw (measured along the outer contour) is 10.4, the height at the base, 6.0; the length of the second digit claw is 6.9, the height, 4.1; the length of the third digit claw is 7.1, the height, 3.6.

## Discussion

*Homotherium* was widespread in the Plio-Pleistocene of Eurasia, Africa, and Americas^11–16^. For a long time, the latest presence of *Homotherium* in Eurasia was recorded in the Middle Pleistocene^17^. A significant event was the discovery of the mandible of the Late Pleistocene *H. latidens* (Owen, 1846) from the North Sea, which is dated to 28 ka^18^. The largest number of Late Pleistocene finds of *Homotherium* is concentrated in North America (more than 30 localities), where they traditionally classified in the species *H. serum* (Cope, 1893)^4,19^.

Genetic analysis of the specimen of *Homotherium latidens* from the North Sea showed its identity to the Late Pleistocene *H. serum* from the North America^20^. In this case, the species name *H. latidens* received priority.

Based on the above, and also taking into account the age of the find, we attribute the Badyarikha mummy to *H. latidens*. This finding is the second evidence of the presence of *H. latidens* in the Late Pleistocene of Eurasia and the first described find from Asia.

The life appearance of *Homotherium* and other sabre-toothed felids has been the subject of long debates^12,21^. In recent years, a number of works devoted to the reconstruction of the muscular system of *Homotherium* have appeared. Signs of hypertrophy of the muscles of the neck and forelimbs were established, and a longer and more massive neck of this sabre-toothed cat compared to Pantherinae was reconstructed^21,22^.

Our study of the general body morphology of the *Homotherium* cub mummy confirmed the results of myological reconstruction for adult individuals. The neck of the described cub is much more massive than the cervical region of *P. leo* (ZMMU S-210286) due to the large mass of muscles of the brachycephalic complex of the mummy. The skull morphology of the studied cub can also be traced on the only known juvenile *Homotherium* skull from Friensenhahn Cave in North America^4^. The observed features are also characteristic of adult individuals, except for the relative zygomatic width of the skull, which in the latter is relatively smaller than in juveniles.

The skeleton of adult *Homotherium* is characterized by a short body and long limbs^12^. The study of the Badyarikha mummy shows that most of the postcranial features of *Homotherium* can be traced already at the age of three weeks.

The length of the forelimbs in the described juvenile *Homotherium* is 18–23% greater than that in the juvenile *P. leo* (ZMMU S-210286); at the same time, the body length of the latter is equal to the dimension of the mummy or exceeds it by approximately 10%. The increased size of the oral fissure (approximately by 15.3%) indicates for wide mouth gape adaptation (it included development of the orbicularis oris muscle, etc.).

The front paw of the juvenile *Homotherium latidens* has a rounded shape. Its width is almost equal to its length, in contrast to lion cubs with their elongated and relatively narrow front paw (Fig. 7). The wide paw, the subsquare shape of its pads, and the absence of a carpal pad are adaptations to walking in snow and low temperatures. The small, low auricles and absence of the carpal pad in Badyarikha *Homotherium* contrast with the taller auricles and normally developed pads in the lion cub. All these features can be interpreted as adaptations to living in cold climate.

## Conclusion

The study of the mummy of the *Homotherium latidens* cub made it possible for the first time to observe its fur, the shape of its muzzle, the shape and position of the auricle, the morphology of the mouth opening and nasal planum. The shape of the front paw of this predator was studied, and the features of the distribution of its muscle mass were established, also for the first time. New information about the juvenile stages of development of the skull and limbs makes it possible to establish the peculiarities of the early postnatal ontogenesis of *Homotherium*. Also, the discovery of *H. latidens* mummy in Yakutia radically expands the understanding of distribution of the genus and confirms its presence in the Late Pleistocene of Asia. Thus, for the first time in the history of paleontological research, the external appearance of an extinct mammal that has no analogues in the modern fauna has been studied directly.

## Data Availability

All data generated or analysed during this study are included in this published article. Please contact Prof. A.V. Lopatin in case of any queries regarding this study.
